# Patient-centred tuberculosis treatment delivery under programmatic conditions in Tanzania: a cohort study

**DOI:** 10.1186/1741-7015-7-80

**Published:** 2009-12-21

**Authors:** Saidi Egwaga, Abdallah Mkopi, Nyagosya Range, Vera Haag-Arbenz, Amuri Baraka, Penny Grewal, Frank Cobelens, Hassan Mshinda, Fred Lwilla, Frank van Leth

**Affiliations:** 1 National Tuberculosis and Leprosy Programme, Ministry of Health and Social Welfare, Dar es Salaam, Tanzania; 2 Ifakara Health Institute, Ifakara, Tanzania; 3 National Institute for Medical Research, Dar es Salaam, Tanzania; 4 Novartis Foundation for Sustainable Development, Basle, Switzerland; 5 KNCV Tuberculosis Foundation, The Hague, The Netherlands; 6 Center for Infection and Immunity, Academic Medical Center, University of Amsterdam, Amsterdam, The Netherlands

## Abstract

**Background:**

Directly observed therapy (DOT) remains the cornerstone of the global tuberculosis (TB) control strategy. Tanzania, one of the 22 high-burden countries regarding TB, changed the first-line treatment regimen to contain rifampicin-containing fixed-dose combination for the full 6 months of treatment. As daily health facility-based DOT for this long period is not feasible for the patient, nor for the health system, Tanzania introduced patient centred treatment (PCT). PCT allows patients to choose for daily DOT at a health facility or at their home by a supporter of choice. The introduction of fixed dose combinations in the intensive and continuation phase made PCT feasible by eliminating the risk of selective drug taking by patients and reducing the number of tablets to be taken. The approach was tested in three districts with the objective to assess the effect of this strategy on TB treatment outcomes

**Methods:**

Cohort analysis comparing patients treated under the PCT strategy (registered April-September 2006) with patients treated under health-facility-based DOT (registered April-September 2005). The primary outcome was the cure rate. Differences were assessed by calculating the risk ratios. Associations between characteristics of the supporters and treatment outcomes in the group of patients opting for home-based DOT were assessed through logistic regression.

**Results:**

In the PCT cohort there were 1208 patients and 1417 were included in the historic cohort. There was no significant difference in cure rates between the cohorts (risk ratio [RR]: 1.06; 95% confidence interval [CI]: 0.96-1.16). In the PCT cohort, significantly more patients had successful treatment (cure or treatment completed; RR: 1.10; 95%CI: 1.01-1.15). There were no characteristics of supporters that were associated with treatment outcome.

**Conclusion:**

The PCT approach showed similar cure rates and better treatment success rates compared to daily health-facility DOT. The results indicate that there are no specific prerequisites for the supporter chosen by the patient. The programmatic setting of the study lends strong support for scaling-up of TB treatment observation outside the health facility.

## Background

Tanzania is one of the 22 high-burden countries with respect to the number of incident tuberculosis (TB) cases[1]. Up to the mid-eighties of the previous century, the annual number of cases identified and treated in the country was manageable, but due to the developing epidemic with the human immunodeficiency virus (HIV), the caseload in Tanzania increased rapidly. Within 5 years, the number of TB cases notified tripled from 5000 to 15,000 between 1980 and 1985. In 2006, Tanzania reported over 62,000 TB cases (157 cases per 100,000 population), while the World Health Organization (WHO) estimated the incidence to be 312 per 100,000 population [1]. Thirty-five percent of smear-positive TB patients were HIV-positive in a recent study [2].

The currently recommended strategy for TB control is the directly observed treatment, short course (DOTS) [3,4]. This strategy includes, amongst others, the daily observation of patients' drug intake at the treating facility during the initial 2 months of therapy. The reason behind this observation is that it will ensure adequate treatment adherence during the period, in which the treatment regimen contains rifampicin which will prevent the selection of *Mycobacterium tuberculosis *strains resistant to this powerful drug [5-8].

The National Tuberculosis and Leprosy Programme (NTLP) of Tanzania changed the standard first-line treatment regimen in 2006 from an 8-month regimen to a 6-month regimen. In the latter, both the intensive phase treatment and the continuation phase treatment contains rifampicin, while in the former rifampicin is only given for 2 months of the intensive phase. The rifampicin-containing regimen throughout the course of treatment has a higher efficacy in situations with a high prevalence of HIV-infection in the general population [9-11]. The WHO recommends the observation of treatment throughout the 6 months of this regimen at least three times a week [3]. This is clearly not feasible at the health facility level for either the patient or the health staff. Initial studies in urban and rural areas of Tanzania showed that decentralized treatment observation by guardians or former TB patients was feasible without a detrimental effect on treatment outcome [12,13]. The main drawback encountered in a possible scale-up of this approach was the need for incentives expressed by the supporters which made the strategy non-sustainable. The experiences from these studies formed the basis of a new approach to the implementation of DOTS in Tanzania in which the observation of treatment intake could be transferred from the health facility to the patient's home and the observer could be changed from a health-care worker to a supporter of choice.

These changes maintain the core principle of daily observation of treatment (DOT) for the full duration of treatment, prevent health facilities from being overburdened and enable the patients to choose a supervision model within the constraints of their daily life. This should have a positive effect on treatment adherence and, subsequently, the efficacy of treatment. The new strategy was labelled 'patient centred treatment' (PCT).

From previous studies it is known that DOT outside the health facility, by either health personnel or laymen, is feasible and can result in treatment outcomes that are similar to those under conventional DOT at the health facility [14-18]. In Nepal, both DOT at the health facility and DOT by family members obtained success rates that met the international target of 85% [17]. However, most of these studies have been designed as randomized clinical trials and/or put restrictions on the type of supporter who can observe treatment intake. Clinical trials have the inherent limitation of coming with increased supervision and monitoring which can have a positive effect of its own on treatment delivery and adherence. Restrictions on supporters complicate the interpretation and replication of results within the context of national control programmes with a wider choice of potential treatment supporters. Thus, there is an urgent need to test the concept of delegating treatment observation outside the health facility under routine programmatic conditions. This need also holds true for fields outside the context of TB where strategies other than health facility-based treatment delivery are being examined, such as home-base care programmes for the delivery of antiretroviral therapy to HIV-infected patients.

The concept of PCT was tested and well perceived by patients and health care professionals when assessed in a qualitative survey. Furthermore, the proposed strategy was seen to be a positive contribution to adherence to therapy [2]. The strategy was formally tested in three districts under programmatic conditions with no restriction on the choice of treatment supporter. The objective of this study was to assess whether favourable treatment outcomes (cure and success) in new patients (that is, those without previous TB treatment) treated under the new strategy were no lower than those in new patients who were treated under the conventional strategy. If so, this would lend strong support to the practice of observing treatment intake outside the health facility by laymen, under programmatic conditions.

## Methods

### Design

Initially, the study was designed as a cohort study comparing treatment outcomes for patients who chose home-based treatment with those who chose health facility-based treatment. This design was based on the results from a community assessment in which patients, if given a choice, were asked what would be their preference for the place of treatment. In this assessment, just over 50% would have opted for home-based treatment [2]. During the implementation of the current study it became clear that a much higher proportion of patients opted for home-based treatment, making a formal comparison of treatment outcomes with patients opting for health facility-based treatment statistically more difficult.

The study design was changed into a comparison of treatment outcomes for patients in the PCT cohort with those in a control cohort of all registered new TB patients in the same facilities during the same period of time a year earlier. This also changed the intervention being tested because three variables in the PCT cohort were different from the control cohort; the possibility of having home-based treatment observation, treatment with fixed dose combinations (FDCs) and a regimen with rifampicin for the continuation phase resulting in 6 months duration instead of 8 months. Possible differences between the cohorts should, therefore, be attributed to the overall intervention of PCT, rather than only the home-based setting of treatment observation.

### Study population and setting

The study was carried out in the Arusha Municipality, the Kahama district (Shinyanga province) and the Mufindi district (Iringa province) of Tanzania. The decision to choose these sites was based on the number of notified smear-positive TB-patients and their representativeness for urban and rural settings in the country. Within each area, all TB treatment facilities implemented PCT for all new patients who were registered in the second and third quarter of 2006. The only inclusion criterion used was that the patient was defined as 'new', indicating that he/she had never received any TB-treatment that lasted longer than 1 month. The control cohort comprised all new patients in the same health facilities registered in the second and third quarter of 2005 who were treated under the conventional DOTS strategy. Smear-positive TB was recorded when two out of three sputum samples (spot, morning, spot) were Ziehl-Neelsen positive. When clinical symptoms were suggestive of TB, but sputum samples were negative, a suggestive chest X-ray could lead to the diagnosis of smear-negative TB.

### Intervention

The intervention tested was PCT, which consisted of three components. First, each patient was given the choice to receive treatment at home observed by a supporter of his/her choice or to receive daily treatment at the health facility observed by health staff. There were no restrictions on the choice of supporter. Second, treatment for all patients was delivered as a FDC rather than the conventional separate tables for each drug. Third, the treatment contained isoniazid, rifampicin, ethambutol and pyrazinamide in the initial phase of 2 months and isoniazid and rifampicin in the continuation phase of 4 months. At any time, patients were allowed to change from home-based treatment to health facility-based treatment or vice versa. There were no interventions related to default tracing and monitoring in the PCT strategy, other than the routine guidelines from the NTLP, making the control cohort and the PCT cohort comparable in this respect.

### Follow-up and data collection

If the patient opted for home-based treatment, he/she was asked to return with the supporter of his choice. The supporter was given instruction by the health care provider on the importance of daily supervision of drug intake, the signs and symptoms of side-effects, and what to do if they occur, and the frequency of the collection of new drugs. Supporters needed to escort the patient to the health facility on a weekly basis in the first 2 months of treatment to collect new drugs, report on the well-being of the patient and to discuss any problem encountered in the support of the patient. The patient was requested to join the supporter at every visit unless too ill to do so. In the remaining 4 months, the visits to the health facility took place twice a month.

Data on the demographics of patients and supporters, drug intake, side effects and laboratory results in the PCT cohort were prospectively recorded in specifically designed registers and cards. Patient and laboratory data in the control cohort were retrospectively retrieved from the TB registers and the patient's treatment cards in the participating health facilities. These routine registers were checked for accuracy (and updated if needed) by the same independent team that collected data in the PCT cohort in order to minimize ascertainment bias. Data collection for both cohorts took place in three rounds from September 2006 to July 2007. Follow-up ended when the last included patient reached the time of treatment completion (April 2007). This meant that the last data collection took place after the treatment outcome of all patients had been recorded.

### Outcome measures and explanatory variables

The primary study outcome was the proportion of new smear-positive patients cured at the end of treatment (6 months in the PCT cohort and 8 months in the control cohort). Secondary outcomes were the proportion of patients with treatment success (cured or treatment completed) at 6 months or 8 months, and the proportion of smear-positive patients with smear conversion at 2 months. In addition, we assessed whether the characteristics of the treatment supporters were associated with treatment outcomes in the group of patients with home-based treatment observation. Cure was defined as a smear-positive patient having a negative sputum smear at the end of treatment and on at least one previous occasion. Treatment completion was defined as a patient having completed treatment but not having a final smear examination. The characteristics of the supporters were categorized based on distribution with the purpose of getting groups of similar size. Age in years was defined as <25, 25-34, 35-44 and ≥45. The relationship with the patient was categorized as 'family member' or 'non family member'. Education was categorized as 'none', 'primary' and 'above primary'. The initially collected variable 'household of supporter' was not used due to the strong collinearity with the variable 'relationship' in the analyses.

### Quality control

All sputum smears during diagnosis and follow-up were confirmed by an independent laboratory technician. Discrepancies between the initial test and the re-reading were resolved by a third reader whose results were final.

### Statistical analyses

Baseline characteristics of the two cohorts were compared using the *χ*^2 ^*test *for binary variables and Student's *t-t*est for continuous variables. Outcome measures in the cohorts were compared by calculating the risk ratio (RR) stratified by district. A combined analysis of all three districts was only performed when there was no effect modification by district as assessed by the Cochran-Mantel-Haenszel test.

Supporter characteristics associated with cure and treatment success in the group of patients with home-based treatment observation were assessed by univariable and multivariable logistic regression. All multivariable analyses included, apart from 'place of treatment' the variable 'district' to incorporate the stratified design in the analyses. Other variables were only included if they were significantly associated with the outcome in univariable analyses with a *P*-value of 0.1 or less. Effect modification by districts in these models was assessed by testing for interactions (multiplicative). Effect modification was assumed statistically significant at the 10% level.

### Missing data and loss to follow-up

Due to an unforeseen logistical problem, the cards designed for recording information on the supporters were not available for the first 205 patients. Unfortunately, not all information could be retrieved at a later stage. This did not introduce a bias as the study started in all the three districts at the same time. Furthermore, the patients' freedom of choice for the type of treatment delivery strategy remained. The missing cards only reduced the power of the study for the analyses assessing the effects of supporter characteristics on treatment outcomes in the PCT cohort.

The missing data were categorized as a separate level in the respective variables in order to include all treatment outcomes in the analyses. Therefore, statistical significant associations were assessed on level the Wald test in the logistic regression models rather than the likelihood ratio test.

### Sample size

Based on the total number of new smear-positive patients who opted for home-based treatment and the corresponding number of patients a year earlier, the power of the study for the primary outcome was an 80% ability to detect a statistically significant difference (α = 5%) of 8.5% when cure in the control cohort was estimated at 70%.

### Ethics statement

The study was approved by the Institutional Review Board of the Ifakara Health Institute in Tanzania. Informed consent was not obtained from the participants as the intervention was based on a change in national treatment guidelines that was applied to all.

### Role of the funding source

The funder of the study was involved in the study design and data interpretation. Data collection and data analysis were performed independently of the funder. The corresponding author had access to all data in the study. The final decision to submit for publication was made by the NTLP of Tanzania.

## Results

**Figure 1:**
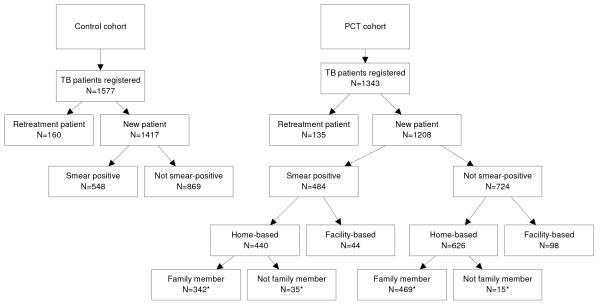
**Patient disposition**. * No information of relationship of treatment supporter for 205 patients.

The study included 1208 new patients in the PCT cohort and 1417 in the control cohort. Of these, 548 (38.7%) and 484 (40.1%) were smear-positive (Figure 1). The proportion of females and the age of the patients were comparable in both cohorts (Table 1). The relative contribution of the districts for both cohorts differed: there were slightly more patients from the urban setting in the PCT cohort (40%) than in the control cohort (30%). Smear-negative patients were slightly under-represented in the PCT cohort compared to the control cohort. None of the analyses showed an interaction by district. Therefore, the outcomes were pooled and analysed for the total study population.

**Table 1:** Baseline characteristics of tuberculosis (TB) patients.

	Historic	PCT	*P*-value
	*n *= 1417*	*n *= 1208*	
District			<0.001
Arusha	394 (27.8)	495 (41.0)	
Kahama	394 (27.8)	329 (27.2)	
Mfindi	629 (44.4)	384 (31.8)	
Female	667 (47.1)	563 (41.9)	0.812
Age group			
<25	292(20.6)	241 (20.0)	0.796
25-34	471 (33.2)	390(32.3)	
35-44	323(22.8)	260(21.5)	
>= 45	323(22.8)	290(24.0)	
Missing	8(0.6)	27(2.2)	
Type of TB			0.020
New smear-positive	548 (38.7)	484 (40.1)	
New smear-negative	473 (33.4)	345 (28.6)	
New EPTB	396 (27.9)	379 (31.4)	

*All data are n (%).

PCT, patient centred treatment; EPTB, extra pulmonary tuberculosis.

### Comparing treatment outcomes between cohorts

Treatment outcomes comparing both cohorts are summarized in Table 2.

**Table 2:** Treatment outcomes.

	Historic	PCT	RR†	95% CI	*P*-value
Smear-positive patients	*n *= 548*	*n *= 484*			
Smear conversion	409 (74.6)	388 (80.2)	1.07	1.01 -- 1.15	0.037
Cure	334 (60.9)	312 (64.5)	1.06	0.96 -- 1.16	0.247
Success	382 (69.7)	398 (82.2)	1.18	1.10 -- 1.26	<0.001
Death	92 (16.8)	59 (12.2)	0.73	0.54 -- 0.98	0.042
Default	28 (5.1)	13 (2.7)	0.53	0.28 -- 0.99	0.055
Transfer out	41 (7.5)	14 (2.9)	0.39	0.22 -- 0.68	0.001
Fail	2 (0.4)	0			
Missing outcome	3 (0.6)	0			
					
All patients	*n *= 1417*	*n *= 1208*			
Success	1015 (71.6)	954 (79.0)	1.10	1.05 -- 1.15	<0.001
Death	249 (17.6)	168 (13.9)	0.79	0.66 -- 0.95	0.012
Default	61 (4.3)	40 (3.3)	0.77	0.52 -- 1.14	0.222
Transfer out	80 (5.7)	41 (3.4)	0.60	0.42 -- 0.86	0.007
Fail	2 (0.1)	0			
Missing outcome	10 (0.7)	4 (0.3)			

*All data are n (%).

† Relative risk comparing PCT cohort with historic cohort.

CI, confidence interval.

#### Smear-positive patients

None of the smear-positive patients in the PCT cohort who had a smear examination at the end of treatment had a positive smear result at the end of treatment, compared to two in the control cohort. This implies that differences in cure rate were driven by the presence of a smear result and the proportion of patients defaulting during treatment. There was no statistically significant difference in the primary outcome (cure) comparing the PCT (64.5%) cohort with the control cohort (60.9%; RR: 1.06; 95% confidence interval [CI]: 0.96-1.16; *P *= 0.136).

Smear-positive patients in the PCT cohort were more likely to have successful treatment (82.2%) than patients in the control cohort (69.7%; RR: 1.18; 95% CI: 1.10-1.26; *P *< 0.001). This was because more patients in the PCT cohort, compared to the historical cohort, completed treatment and had a final smear examination performed at the end of treatment (necessary in order to confirm cure).

Significantly more smear-positive patients in the PCT cohort had a recorded smear conversion at 2 months (80.2%), than in the control cohort (74.6%; RR: 1.07; 95% CI: 1.01-1.15; *P *= 0.021). This difference was driven by the fact that a larger proportion of patients in the control cohort (20.1%) died, transferred, defaulted or did not have a sputum smear performed compared to the PCT cohort (15.5%). Comparing the proportion of patients with smear conversion only for those patients who were present at month 2, and had had a smear examination, did not reveal statistically significant changes between the PCT cohort (93.4%) and the historic cohort (94.9%; *P *= 0.359).

#### All new patients

A comparison of the treatment outcomes for all new patients included in the two cohorts showed a similar finding of a higher success rate in the PCT cohort (79%) compared to the control cohort (71.6%); RR: 1.10; 95% CI: 1.05-1.15; *P *< 0.001).

### Comparing treatment delivery strategies in the PCT cohort

New smear-positive patients who opted for home-based treatment observation were more likely to be cured compared to patients having treatment observation at the health facility (RR: 2.37; 95% CI: 1.27 - 4.43; *P *= 0.007; Table 3). This was seen in all districts, although the effect was larger in Arusha than in Kahama and Mufindi which, however, did not lead to a statistically significant effect modification in any of the statistical models. Adjusting the effect of home-based treatment for these differences in districts did not change the results (RR: 2.31; 95% CI: 1.22 - 4.38; *P *= 0.010).

**Table 3:** Factors associated with cure smear-positive patients in patient centred treatment cohort.

	Univariable			Multivariable		
	OR	95% CI	*P*	OR	95% CI	*P*
Treatment observation*						
HF-DOT	1			1		
HB-DOT	2.37	1.27 -- 4.43	0.007	2.31	1.22 -- 4.38	0.010
District						
Kahama	1			1		
Arusha	1.38	0.87 -- 2.20	0.169	1.27	0.79 -- 2.04	0.315
Mufindi	1.18	0.74 -- 1.89	0.482	1.04	0.65 -- 1.70	0.849
						
Supporter characteristics†						
Sex						
Male	1					
Female	1.19	0.75 -- 1.89	0.450			
Relationship						
Non-family member	1					
Family member	1.09	0.46 -- 2.61	0.843			
Age						
< 25 years	1					
25 -- 34 years	1.06	0.50 -- 2.24	0.879			
35 -- 44 years	0.96	0.42 -- 2.15	0.912			
>= 45 years	0.84	0.38 -- 1.85	0.670			
Education						
None	1					
Primary education	1.74	0.92 -- 3.27	0.087			
Above primary education	1.64	0.69 -- 3.89	0.258			

* In all new smear-positive patients (*n *= 484)

† In HB-DOT group (*n *= 440)

HF, health facility; HB, home-based; DOT, directly observed treatment; CI, confidence interval; OR, odds ratio.

Treatment success in the entire PCT cohort was also more likely to be reported by patients who choose home-based treatment (RR: 4.19; 95% CI: 2.91 - 6.05; *P *< 0.001). Again, the effect was larger in Arusha than in the other two districts but did not lead to significant effect modification. Adjustment did not alter the effect of home-based treatment (RR: 4.11; 95% CI: 2.82 - 6.00; *P *< 0.001).

### Effect of supporters on treatment outcomes

Of the supporters, 76% were family members (including spouses), 5% were non-family members - for 19%, no information on the relationship was available. None of the supporter characteristics was associated either with cure of smear-positive patients (Table 3) or treatment success for any of the patients (Table 4). The cure and treatment success rates in patients who had a family member as a supporters were as high as patients who had a non-family member as a supporter.

**Table 4:** Factors associated with success all patients in patient centred treatment cohort.

	Univariable			Multivariable		
	OR	95% CI	*P*	OR	95% CI	*P*
Treatment observation*						
HF-DOT	1			1		
HB-DOT	4.19	2.91 -- 6.05	< 0.001	4.11	2.82 -- 6.00	< 0.001
District						
Kahama	1			1		
Arusha	0.52	0.37 -- 0.74	<0.001	1.64	1.15 -- 2.33	0.006
Mufindi	0.49	0.36 -- 0.69	<0.001	0.81	0.57 -- 1.16	0.262
						
Supporter characteristics†						
Sex						
Male	1					
Female	0.99	0.67 -- 1.47	0.974			
Relationship						
Non-family member	1					
Family member	1.05	0.46 -- 2.39	0.910			
Age						
< 25 years	1					
25 -- 34 years	0.70	0.35 -- 1.43	0.332			
35 -- 44 years	0.92	0.43 -- 1.98	0.845			
>= 45 years	0.50	0.25 -- 1.05	0.067			
Education						
None	1					
Primary education	1.61	0.92 -- 2.85	0.098			
Above primary education	0.97	0.49 -- 1.96	0.950			

* In all new patients (n = 1208)

† In HB-DOT group (n = 1066)

OR, odds ratio; CI, confidence interval; HF, heath facility; HB, home-based.

## Discussion

This study showed that treatment outcomes in a cohort of new TB patients receiving treatment under the PCT strategy were comparable to treatment outcomes in patients who received treatment with the conventional health facility-based DOT and administration of loose drugs. The higher success rate in the PCT cohort is driven by a larger proportion of patients completing their treatment. This finding is not easy to interpret. It might be just an effect caused by the fact that the treatment was for 2 months less. For the verifiable outcome cure, there is no difference between the historical cohort and the PCT cohort. In addition, the study did not identify any supporter characteristic that influenced the achievement of cure or treatment success in patients who had their treatment observed at home.

These findings are in line with the results from other studies performed in a variety of settings. A cluster-randomized trial in Nepal showed that 89% of the patients who had their treatment intake observed by a family member had a treatment success. This was comparable with the 85% success in patients who were supervised by a community health worker and higher than the treatment targets from the WHO (85%) [17]. A retrospective study in Thailand assessed treatment outcomes in patients who accepted direct observation of treatment by staff of the health facility, a village health worker or a family member. Ninety percent of the patients opted for observation by a family member and 10% for the health staff of the facility. Only one of the 216 patients chose observation by a village health worker. The success rate was higher in patients observed by a family member (87%) compared to patients observed by health staff (76%) [15]. A randomized trial in Swaziland reported that treatment observation by family members obtained similar success rates in TB patients as did treatment observation by community health workers (66% and 68%, respectively) [18]. Similarly, a randomized trial in Pakistan did not find any differences in treatment success rates in patients observed by family members (62%), observation at the health facility (67%) or self-administration (62%) [19].

Despite these findings, the ultimate proof of principle for the efficacy of home-based treatment observation is not given by the short-term cure rates or success rate as assessed in this and other studies. Only when, in settings with home-based DOT, the relapse rates do not increase (and preferentially decrease due to improved adherence) can home-based DOT be regarded as effective.

The concept of treatment observation by family members is still controversial within the TB community. The WHO treatment guidelines state that family members should, in general, not be considered as treatment supporters [3]. However, in the updated guidelines on community involvement in TB care, there is a shift towards the idea that there is a need to provide 'an increased range of treatment support options', although this statement was mainly geared at TB patients co-infected with HIV [20].

Despite the above mentioned results of the PCT approach, the success rate in Tanzania remains below the 85% target set by WHO. This relatively low success rate is mainly driven by the death rate. It is more than likely that this rate is largely a result of co-infection with HIV. It is known that HIV-associated death in TB patients is generally seen at the start of TB-treatment. Even the PCT approach will not have a major impact on these events, which is reflected in a death rate of 12%.

There are several arguments formulated by those who are against the use of family members as treatment supporters. First, family members are not always able to be firm on treatment intake, especially if the patient suffers side effects or if the social context makes it difficult or inappropriate to do so [21]. Second, the design of most studies showing similar, or better, treatment outcomes with observation by family members than by health workers actually test multifaceted interventions including increased monitoring, supervision and training. In the Nepal study, there were frequent visits to the households by research staff in order to monitor the implementation and to collect data. In Thailand, clinic staff were strongly involved in the identification of a treatment supporter and special boxes were prepared in order to facilitate treatment delivery at home [15]. In Senegal, the intervention was clearly multifaceted, including improved communication between health staff and patients and increased training and supervision, making it impossible to tease out the effect of decentralized treatment observation [22]. Third, treatment observation outside the health facility is, in practise, more frequently self administration of treatment. This was shown in a study by a study in Thailand where 11% of the health personnel did not perform any treatment observation, compared to 23% of community members and 35% of family members [23].

The major limitation of the study was the change in design which meant that the present study was not able to adequately test the independent effect of the type of treatment delivery. Instead, it tested a multifaceted intervention. Each part of this intervention might have contributed to the positive findings. In the PCT cohort, all drugs were taken as FDC during the full treatment period. In the control cohort, FDC was only available for isoniazid and rifampicin during the intensive phase. All other drugs were administered as loose drugs. Drug delivery by FDCs might be associated with an improved adherence to therapy [24]. At the same time, the treatment regimen was changed to include rifampicin throughout the duration. An additional 4 months of this potent drug might positively influence the cure rate in the PCT cohort. However, the additional efficacy of this treatment regimen over the conventional regimen used in the control cohort is based largely on the relapse rates and not on the initial cure rates. If the new regimen had an effect on the outcome it was most likely due to its shorter duration and, therefore, the possible higher completion rates, rather than an increased drug efficacy. However, the total intervention will be rolled-out in the rest of the country making the individual contributions of the multifaceted intervention towards the overall positive effect less important. The important finding, that the type of treatment supporter does not influence treatment outcome, makes this nationwide scale-up of the intervention more feasible.

Another limitation was the absence of demographic information for a considerable number of treatment supporters. As mentioned, this did not introduce a bias because of the concurrent start of the study in the districts and the retaining of the freedom of choice for the type of treatment delivery strategy. There is also a marked absence of HIV data form the enrolled patients. At the time of the study (2005 for the historical cohort and 2006 of the PCT cohort), routine HIV testing for newly registered patients had not been fully implemented. Collection of this information for inclusion in statistical models would lead to a bias, given the large proportion of patients who would not have this information. A comparison of this information between the two cohorts would also be not possible due to the increase in routine testing between 2005 and 2006.

There is some indication that ascertainment bias might have influenced the results in Arusha. In this district case notification increased considerably during the study and there was, also, a significantly higher probability of cure in the PCT cohort. This may indicate improved diagnosis and/or microscopy. It could also be a result of the quality control measures that were imposed in the PCT cohort but not the historical cohort. However, adjusting the multivariable models did not give any change in the point estimates or the confidence intervals, making this potential bias of little value.

Acceptable and feasible treatment observation strategies are highly context specific [25]. In Tanzania, home-based treatment observation by a supporter of choice is widely appreciated and does not lead to an increase in unfavourable treatment outcomes compared to conventional treatment delivery strategies. It allows patients to rest and recover, as daily health facility DOT places considerable physical strain on patients. Being at home also made it possible for the patient to engage in productive activities and reduce the cost of travel associated with the daily health facility visits. At the same time, it could ease the burden on health facilities [2]. Studies from Malawi and Australia have shown that, in a decentralized setting, adherence to treatment can be as high as seen in health-facility based treatment observation [16,26]. However, it is important to assess adherence to treatment in the setting of Tanzania.

The programmatic condition in which this study was carried out lends strong support to the scaling up of the PCT strategy in Tanzania. This must coincide with rigorous supervision of the health facilities implementing the strategy. Furthermore, follow-up of this first cohort of patients treated under the PCT strategy will give much needed data on the risk of relapse and the resistance patterns in patient who need retreatment: two factors that determine the long-term efficacy of the intervention.

The positive effect of home-based treatment in a programmatic setting found in this study can be of value for areas outside the context of TB. HIV-programmes in especially resource-poor areas are experimenting with such an approach [27,28]. The findings that home-based treatment can be achieved under programmatic conditions might give the much needed impetus to move from smaller projects to a more general implementation within national treatment programmes.

## Conclusions

The PCT approach showed similar cure rates and better treatment success rates compared to daily health-facility DOT. The results indicate that there are no specific prerequisites for the supporter chosen by the patient. The programmatic setting of the study lends strong support for scaling-up of TB treatment observation outside the health facility.

## Abbreviations

CI: confidence interval; DOT: directly observed therapy; DOTS: DOT, short course; FDC: fixed dose combination; NTLP: National Tuberculosis and Leprosy Programme; PCT: patient centred treatment; RR: risk ratio; TB: tuberculosis; WHO: World Health Organization.

## Competing interests

VH was a full time employee of Novartis Foundation for Sustainable Development during the entire study. At the time of the study design, PG was also a full time employee of the Novartis Foundation for Sustainable Development. None of the other authors declared a competing interest.

## Authors' contributions

SE, NR and FL participated in the design and implementation of the study and provided critical contributions to the drafts of the manuscript. AM and AB collected and analysed the data and provided critical contributions to the drafts of the manuscript. HM supervised the data collection and analysis. VH, PG and FC participated in the design of the study and provided critical contributions to the drafts of the manuscript. FvL participated in the design of the study, assisted in data analysis and wrote the first and all consecutive drafts of the manuscript.

## Pre-publication history

The pre-publication history for this paper can be accessed here:

http://www.biomedcentral.com/1741-7015/7/80/prepub
